# An Unusual Presentation of Endometrial Cancer with Bilateral Adrenal Metastases at the Time of Presentation and an Updated Descriptive Literature Review

**DOI:** 10.1155/2019/3515869

**Published:** 2019-12-23

**Authors:** Maisie Ryan, Alexandros Laios, Darshana Pathak, Michael Weston, Richard Hutson

**Affiliations:** ^1^Department of Gynaecologic Oncology, St. James's University Hospital, Leeds, UK; ^2^Department of Cellular Pathology, St. James's University Hospital, Leeds, UK; ^3^Dapartment of Radiology, St. James's University Hospital, Leeds, UK

## Abstract

In endometrial cancer (EC), adrenal metastases are rare indicating advanced disease. We report an unusual presentation of EC with solitary adrenal metastases at the time of diagnosis and provide with an updated literature review. A 68-year-old woman was referred with postmenopausal bleeding of several weeks' duration. Imaging revealed a heterogenous uterine mass and bilateral malignant adnexal masses. Hysteroscopy, endometrial biopsies, and radiological guided biopsies of the adrenal masses confirmed poorly differentiated EC. A PET-CT reported both adrenal metastases being hypermetabolic and suspicious for malignancy. The patient received six neoadjuvant chemotherapy cycles with Carboplatin and Paclitaxel. A repeated CT scan confirmed size reduction for both primary tumour and metastases. The adrenal metastases were no longer PET-avid on repeat PET-CT scan. The patient received a course of hormonal treatment and as per adrenal MDT, she underwent total laparoscopic hysterectomy and bilateral salpingo-oophorectomy followed by bilateral retroperitoneal laparoscopic adrenalectomy two months later. The patient remains asymptomatic on maintenance hydrocortisone 18 months post diagnosis. This is the first report of solitary synchronous adrenal metastases in a patient with EC. Central MDT review is key in providing individualised treatment recommendations of such rare entity.

## 1. Introduction

Endometrial cancer (EC) is the most common gynaecological malignancy with an average of 9,000 new cases diagnosed in the UK each year [1]. Between 1995 and 2010, the incidence of EC in the UK increased by 43% resulting in a 14% associated death increase [2]. In the UK, 18% of women present with advanced stage 3-4 disease, which carry a 5 year survival rate of 60% and 29%, respectively [3]. Approximately 7% of patients have metastases at diagnosis [1, 3]. Adrenal metastases in EC are rare, indicating advanced stage disease [4]. Surgery is the milestone of treatment, consisting of total hysterectomy and bilateral salpingo-oophorectomy, but the role of systemic lymphadenectomy and adjuvant treatment are still debated. If at presentation, the tumour is not amenable to operative management, then the approach must be tailored, and chemotherapy or systemic hormonal therapy should be considered. Here, we report an unusual presentation of EC with solitary adrenal metastases at the time of diagnosis and an update of the current literature.

## 2. Case Presentation

**Figure 1:**
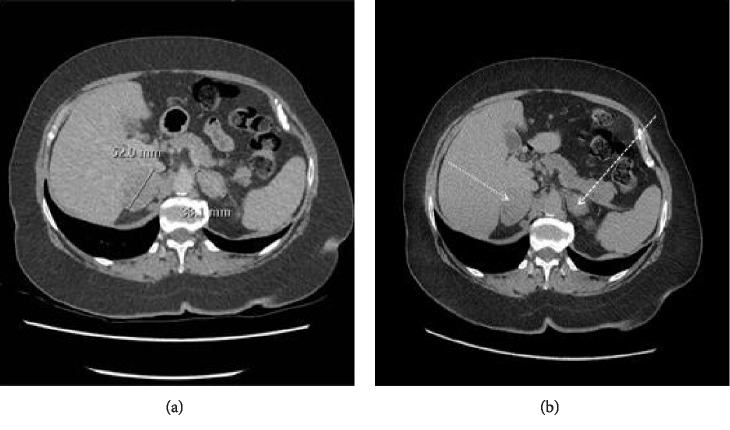
Initial pre- and postcontrast CT image (axial) demonstrating bilateral adrenal metastases (arrows). Nonenhanced CT imaging is actually very helpful because it can dismiss the lesions as benign adenomas.

A 68-year-old Caucasian woman was referred into secondary care by her GP with abdominal bloating and postmenopausal bleeding of several weeks' duration. She was otherwise fit and well. As per NICE guidelines she had an urgent abdominopelvic ultrasound [5], which revealed a 32 × 39 mm lobulated heterogeneous mass from the posterior wall of the endometrial cavity and an incidental 61 × 29 × 59 mm well-defined mass, superior to the right kidney, which was suspicious for an adrenal mass. Her physical examination was unremarkable. Urgent cross-sectional imaging and direct visualisation of the uterine cavity was advised. The patient underwent hysteroscopy, and endometrial biopsies, which showed a grade 3 endometrial adenocarcinoma. A pelvic MRI confirmed the presence of a large endometrial tumour extending into the posterior myometrium and a 5.9 cm complex mass in the Pouch of Douglas. The patient had a full body CT, which showed bulky bilateral adrenal masses, which were not in keeping with benign adrenal adenomas (Figure 1).

**Figure 2:**
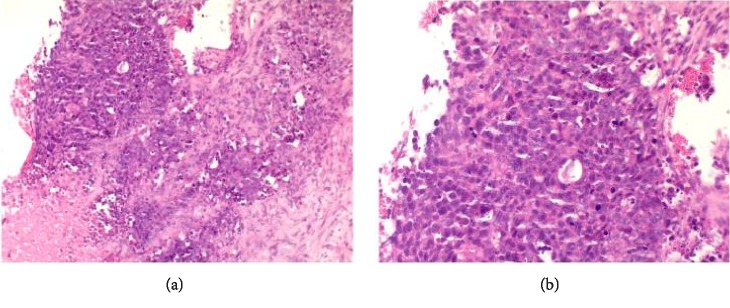
(a) H&E stained section of core biopsy of adrenal gland (×20 magnification). (b) H&E stained section of core biopsy of adrenal gland (×40 magnification). As this was a core biopsy, there was little tumour in p53 stained slide. No pole or MSI profiles were performed.

**Figure 3:**
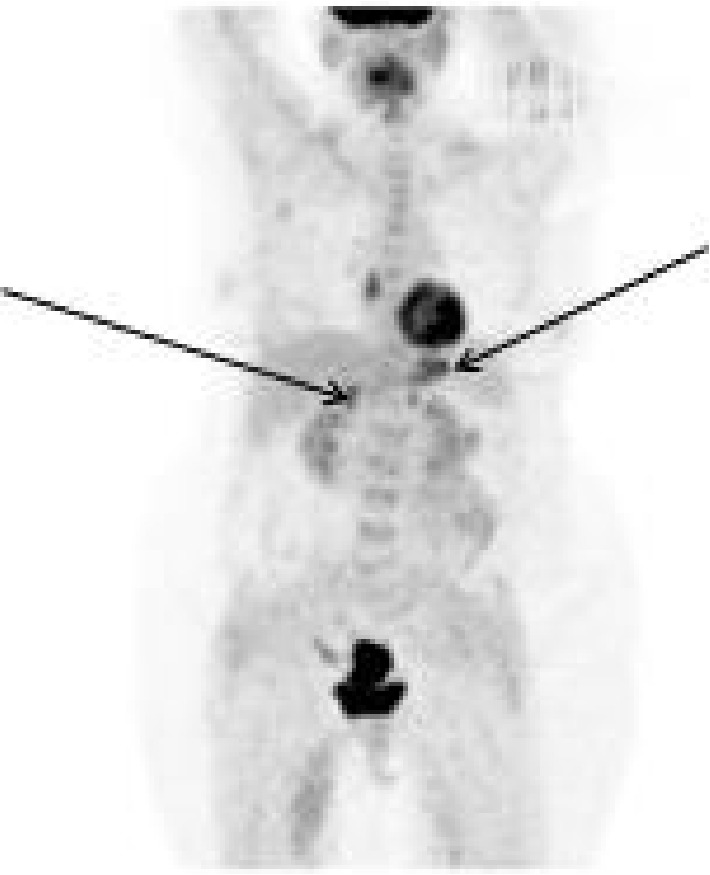
PET-CT scan prior to treatment initiation demonstrating hypermetabolic bilateral adrenal nodules (black arrows) with increased FDG uptake.

The case was discussed at the central multidisciplinary team meeting (MDT) and the recommendation was to have radiological guided biopsy of the adrenal mass. The histology of the right adrenal mass showed a poorly differentiated adenocarcinoma in keeping with primary endometrial cancer (Figure 2). A panel of representative immunohistochemistry was undertaken, but unfortunately in the antibodies of interest, the tumour was cut out. Therefore, consensus was reached by two external pathologists who agreed on the identical morphology between the adrenal tumours and the primary endometrial tumour. To resolve initial concerns about two synchronous primary lesions, a PET-CT scan had been requested, which reported the two adrenal metastases being hypermetabolic and suspicious for malignancy, showing increased FDG uptake (Figure 3). A diagnosis of Stage 4b, Grade 3 EC with adrenal metastases was made, and the patient was referred to the medical oncologists for consideration of neoadjuvant chemotherapy.

**Figure 4:**
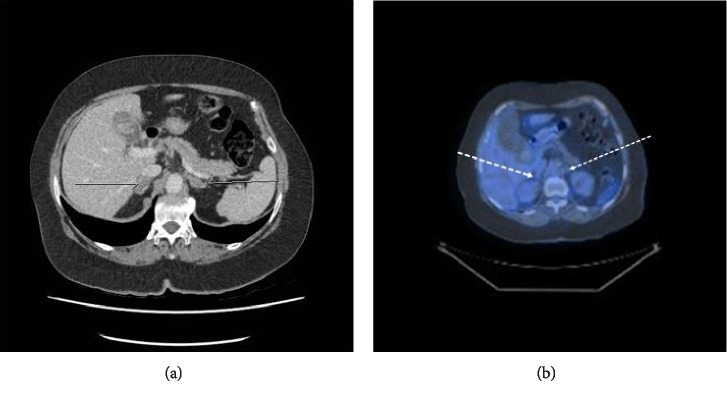
(a) Axial noncontrast CT image following six cycles of primary chemotherapy demonstrating good response with both adrenal metastases decreased in size (white arrows). (b) The adrenal metastases were no longer PET-avid.

The patient commenced on neoadjuvant chemotherapy with Carboplatin and Paclitaxel. She tolerated the chemotherapy reasonably well. Following her third cycle, she developed neutropenic sepsis and was treated with intravenous antibiotics and G-CSF injections. She then had a repeat CT scan, which showed size reduction in the primary tumour and metastases. A decision for further three cycles of Carboplatin and Paclitaxel with a 20% dose reduction was made as it was still felt that surgical intervention would be of no benefit at that point. Nevertheless, the option of palliative radiotherapy was discussed with the patient if she were to develop heavy vaginal bleeding. Following six chemotherapy cycles, a repeat CT showed further good response of the primary tumour and the adrenal metastases (Figure 4(a)). She was rediscussed at the Gynaecological Oncology MDT and a repeat PET-CT was requested. The uterine mass was still prevalent, but the adrenal metastases were no longer PET-avid (Figure 4(b)). No extrauterine disease was identified. She was commenced on alternating hormonal therapy with Megace/Tamoxifen three-weekly. The adrenal MDT recommended a bilateral adrenalectomy following pelvic surgery. The patient underwent a total laparoscopic hysterectomy, bilateral salpingo-oophorectomy and adhesiolysis followed by a bilateral retroperitoneal laparoscopic adrenalectomy two months later. The histology of the adrenal glands showed complete pathological chemotherapy response and no evidence of viable metastatic cancer in either adrenal gland. The patient was reviewed in clinic three and six months after her adrenalectomy with no clinical evidence of recurrent disease. She has been discharged from the endocrine clinic and remains on maintenance hydrocortisone.

We searched the MEDLINE and EMBASE databases for articles published from inception to April 2019 using medical subject heading (MeSH) terms. Key terms included “endometrial cancer” and “adrenal metastases”. The search was limited to the words “humans and adult female”. Publications were cross-referenced from reference lists to obtain additional citations. Only case series published in English language but with no geographical restrictions were included in the literature review. The electronic search initially yielded 14 citations. All reports were published in English language. Four studies were unrelated after screening titles. Nine publications were finally included in the literature review. The main characteristics of those case report studies are shown in Table 1.

## 3. Discussion

In EC, the most common sites of metastatic spread include local invasion to the cervix, vagina, bowel, and bladder or lymphatic spread via pelvic and para-aortic lymph nodes [4]. Solitary adrenal metastases are rare. We identified nine cases within the literature, which describe recurrent metastatic spread to the adrenal glands. These metastases were picked up months to years after the original EC presentation and surgical management [6–13]. A case of dedifferentiated EC with metastatic spread to the cerebellum and adrenal gland at the time of presentation has been also recently reported [14]. To the best of our knowledge, this is the first report of EC with solitary adrenal metastases at the time of diagnosis.

Anatomically, the afferent lymphatic drainage of EC depends on the site of primary tumour, but can often include the para-aortic lymph nodes. Interestingly, the adrenal glands also drain lymph into the para-aortic lymph nodes. Nevertheless, drainage from both organs would be via afferent lymphatics. Therefore, metastatic spread to the adrenals is likely to involve both lymphatic and haematological routes, through efferent lymphatic drainage into the subclavian vein [15]. It is equally striking in the rarity of our case that the adrenals represented the sole solitary metastases. Modern oncology supports the “seed and soil” hypothesis, in which the “seed” represents the cancer cells and the “soil” represents tissues which can promote the growth of that cancer cell through the appropriate receptors and growth factors. This may provide a plausible explanation as only 50% of metastases can be explained by anatomical features such as blood and lymph supply alone [16]. Mismatch repair protein deficient endometrioid adenocarcinomas have been recently reported to metastasize to adrenal gland and lymph nodes [17].

Adrenal metastases often run asymptomatic, but patients may present with adrenal insufficiency if most of the adrenal gland is replaced or destroyed [18]. Metastatic tumours are often misdiagnosed as primary adrenal tumours. CT and MRI are rather limited to define the nature of an adrenal mass. PET scan albeit not recommended for routine preoperative EC staging in the NHS outside a clinical trial, has been reported to successfully identify adrenal metastases [19]. Nevertheless, a fine-needle aspiration biopsy is equally advisable.

Advanced EC is considered a systemic disease with a high rate of relapse underlining the need for an effective systemic therapy. Currently, there are no guidelines for treating patients with solitary adrenal metastases. Therefore, the recommended treatment is still unclear and must be individualised. Recent studies have demonstrated the feasibility of adrenalectomy for solitary adrenal metastases and not just primary adrenal tumours, which could lead to a longer survival in some patients [6, 20]. Neoadjuvant chemotherapy and delayed primary surgery may be an alternative approach in the treatment of selected patients with advanced endometrial cancer who are considered poor candidates for upfront surgery. NACT would be usually reserved for patients where it would be expected that primary debulking surgery would not achieve complete macroscopic resection.

Commonly used regimens incorporate platinum agents including cisplatin and carboplatin in conjunction with either an anthracycline e.g. Doxorubicin or ataxane e.g. Paclitaxol. The optimal chemotherapy schedule of carboplatin and paclitaxel (CP) is derived from the similar responses of EC to epithelial ovarian cancer. This is clearly superior to cisplatin and doxorubicin in terms of its reduced toxicity and deliverability. It is debatable whether there would be justification to carry out a clinical trial of these regimes.

This is the first report of solitary synchronous adrenal metastases in a patient with EC. Considering the EC high prevalence, metastatic spread to the adrenal glands remains rare and poorly understood with only a handful of cases identified through a literature search. Going forward, it would be interesting to explore what it is about adrenal gland tissue which promotes the growth of EC. This knowledge could lead to the identification of new biochemical targets for cytotoxic therapy.

## Figures and Tables

**Table 1:** Summary of case reports identified on literature review.

Patient age	Stage at presentation	Histology	Sites of metastases	Time from presentation to metastases	Author
76	IV	Adenocarcinoma	Acetabulum	0 months	Baron et al. [6]
			Right adrenal	9 months	
62	I	Adenocarcinoma	Lung	7 years	
			Bilateral adrenal	9 years	
77	I	Clear-cell	Cerebral	26 months	Nakano et al. [7]
		Squamous carcinoma	Left adrenal	Post mortem	
		Adenocarcinoma	Posterior mediastinum		
			Oesophagus		
			Hilar lymph nodes		
			Lung		
60	II	Papillary serous	Lung	3 years	Lubana et al. [9]
			Right adrenal	6 years	
58	I	Dedifferentiated	Left adrenal	1 year	Mouka et al. [10]
62	I	Adenocarcinoma	Left adrenal	3 months	Ladwa et al. [11]
55	III	Adenocarcinoma	Pelvic lymph nodes	0 months	Izaki et al. [13]
			Right adrenal	14 months	
62	III	Adenocarcinoma	Pelvic lymph nodes	0 months	Choi et al. [12]
			Left adrenal	10 months	
			Liver	13 months	
75	I	Adenocarcinoma	Bilateral adrenal mets	7 months	Zaidi et al. [8]
67	Stage IV	Dedifferentiated	Cerebellum	0 months	Berretta et al. [14]
			Right adrenal gland		
